# Beyond Protein Synthesis; The Multifaceted Roles of Tuberin in Cell Cycle Regulation

**DOI:** 10.3389/fcell.2021.806521

**Published:** 2022-01-14

**Authors:** E. Fidalgo da Silva, J. Fong, A. Roye-Azar, A. Nadi, C. Drouillard, A. Pillon, L. A. Porter

**Affiliations:** Department of Biomedical Sciences, University of Windsor, Windsor, ON, Canada

**Keywords:** tuberin, TSC, cell cycle, mTOR, cell growth, tuberous sclerosis complex

## Abstract

The ability of cells to sense diverse environmental signals, including nutrient availability and conditions of stress, is critical for both prokaryotes and eukaryotes to mount an appropriate physiological response. While there is a great deal known about the different biochemical pathways that can detect and relay information from the environment, how these signals are integrated to control progression through the cell cycle is still an expanding area of research. Over the past three decades the proteins Tuberin, Hamartin and TBC1D7 have emerged as a large protein complex called the Tuberous Sclerosis Complex. This complex can integrate a wide variety of environmental signals to control a host of cell biology events including protein synthesis, cell cycle, protein transport, cell adhesion, autophagy, and cell growth. Worldwide efforts have revealed many molecular pathways which alter Tuberin post-translationally to convey messages to these important pathways, with most of the focus being on the regulation over protein synthesis. Herein we review the literature supporting that the Tuberous Sclerosis Complex plays a critical role in integrating environmental signals with the core cell cycle machinery.

## Introduction

How a cell adapts to enable cell growth and cell proliferation, or to maintain homeostasis, has been the focus of decades of research with still many fundamental questions without answers. Cell growth occurs when a cell accumulates size and mass in preparation for division. This includes processes involved in protein synthesis, organelle biogenesis, and lipid biosynthesis. Cell proliferation constitutes all the subsequent cellular functions that contribute to the rate at which a cell divides into two daughter cells (Jorgensen and Tyers, 2004). Regulatory cyclin proteins and their catalytic cyclin dependent kinase (CDK) partners are activated in a temporal manner thereby phosphorylating a wealth of downstream substrates to progress cells through the phases of the cell division cycle (Malumbres, 2014). Inhibition of cyclin-CDKs in response to extrinsic and intrinsic signal transduction pathways halts cell cycle progression either temporarily or permanently, thereby enabling cells to repair damage, to alter cell fate, to accumulate proteins and resources or trigger programs of apoptosis or autophagy. Classic tumor suppressors like p53 and the retinoblastoma protein (Rb) play a critical role in these processes, but the literature describes several other proteins with the ability to control cell cycle progression and monitor the surrounding environment that are much less well understood. In this review we discuss the current understanding of the interplay between the cell cycle and the Tuberous Sclerosis Complex (TSC) with a specific focus on one component of the TSC, the protein Tuberin.

## Overview of Cell Cycle Regulation by Cyclin-CDK Complexes

The cell cycle can be grouped into two distinct series of events: interphase and mitosis. Interphase involves cell growth and DNA duplication while mitosis regulates the division of a cell into two daughter cells. Interphase can be further broken down into three phases: gap 1 (G1), synthesis (S), and gap 2 (G2). Mitosis is comprised of five phases: prophase, prometaphase, metaphase, anaphase, and telophase. The cell moves from phase to phase based on the production and destruction of cyclin proteins which bind to their catalytic CDK partner (Pines and Hunter, 1989).

**FIGURE 1:**
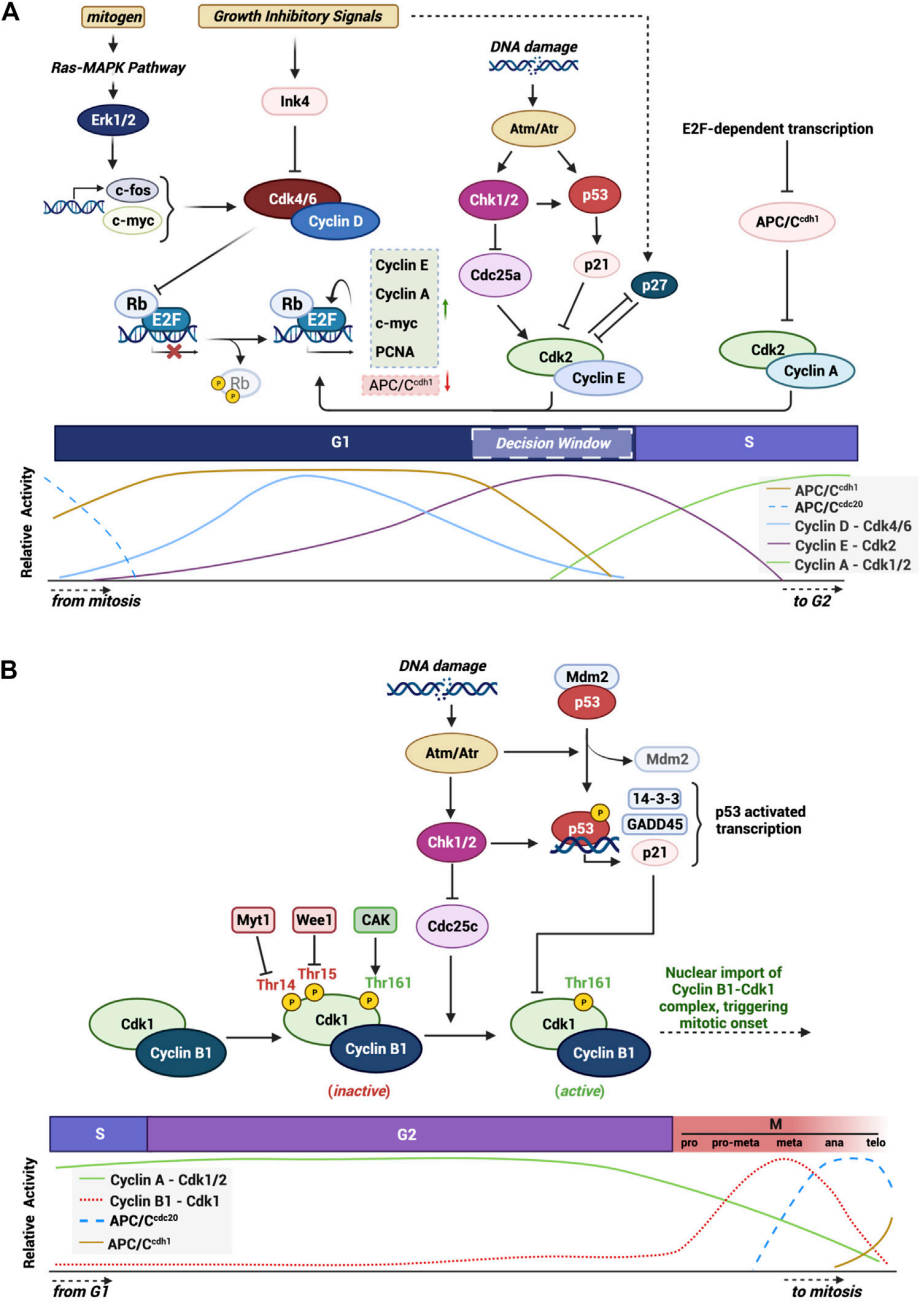
Graphical summary of mechanisms and checkpoints that modulate the activity of cell cycle progression. Highlight of the various integrated pathways and signals that control proper cell cycle progression through the activity of main CDKs and their CKIs. **(A)** G1/S checkpoint and the major proteins and pathways in the progression of the cell through the G1 phase into S phase. **(B)** Specific highlight of the G2/M checkpoint and the specific signals and proteins involved in triggering mitotic onset. Created with BioRender.com.

Movement through early G1 phase of the cell cycle is driven by accumulation of Cyclin D and the binding to CDK4/6. CDK4/6 phosphorylate and inactivate Rb, an adapter protein which represses the activity of transcription factors such as E2F (Sherr, 1994; Weinberg, 1995; Topacio et al., 2019). E2F-dependent transcription creates a positive feedback loop driving transactivation of G1 cyclins, Cyclins E and A, which complex with, and activate, CDK2. During G1 phase, pre-replication complexes form where the origin recognition complexes (ORC1-6) recognize and bind to DNA replication origins. Recruitment of the chromatin licensing and DNA replication factor 1 (CDT1), along with the proteins CDC6 and the minichromosome maintenance complexes (MCM2-7) poise the DNA to unravel and initiate DNA synthesis. CDK2 activation in late G1 triggers a host of events including the assembly of the pre-initiation complex and subsequent disassembly of CDC6 and CDT1 permitting the triggering of MCM helicase activity. This loads primase and other DNA polymerases onto the DNA to kick cells into S phase (Bochman and Schwacha, 2009; Limas and Cook, 2019). The events occurring through DNA synthesis have been extensively studied and will not be detailed in this review (Takeda and Dutta, 2005; Kelly and Callegari, 2019). At the end of S phase the replicative helicase is targeted for ubiquitin-mediated degradation, DNA is packaged into histones and the cell enters G2 phase (Zhang and Li, 2021) (see summary Figure 1A).

The main cyclin protein responsible for transition through G2 and into mitosis in mammalian cells is Cyclin B1. Cyclin B1 is encoded by the *CCNB1* gene on chromosome 5q13.2 and forms a 62 kDa protein capable of being phosphorylated on numerous residues. Cyclin B1 contains two important domains: cyclin box region (aa210-348) that mediates its binding to the G2/M CDK, CDK1 (Smits and Medema, 2001; Petri et al., 2007), and a cytoplasmic retention signal (CRS: aa88-154) which harbors a nuclear export signal (NES: aa142-151) within this region (Smits and Medema, 2001; Petri et al., 2007). Each of these regions are important in regulating the sub-cellular localization of the protein. During late S phase and early G2 phase, the cyclin box domain of Cyclin B1 associates with CDK1 to form an inactive complex (Pines and Hunter, 1989). In late G2 the levels of Cyclin B1 reach a peak and CDK1 is activated via a series of phosphorylation and dephosphorylation reactions (Porter and Donoghue, 2003). Once formed, the complex Cyclin B1-CDK1 triggers a rapid nuclear movement of Cyclin B1 via an unclear mechanism but which involves the autophosphorylation of S126 and S128 within the CRS region of Cyclin B1 thereby preventing the exportin CRM1 from binding to the NES and collectively supporting the nuclear accumulation of the complex (Pines and Hunter, 1989; Hagting et al., 1999; Gavet and Pines, 2010). The activated complex triggers a wave of phosphorylation along the way involving both cytoplasmatic and nuclear substrates including microtubules (Andersen et al., 1997), caspases (Allan and Clarke, 2007) and critical mitotic kinases such as Aurora B and Haspin (Sirri et al., 2002; Qian et al., 2015). During prophase of mitosis, the Cyclin B1-CDK1 complex regulates events responsible for nuclear envelope breakdown, chromosome condensation and mitotic spindle assembly (Jackman et al., 2003). The Cyclin B1-CDK1 activity promotes activation of the APC/C^CDC20^ which creates a negative feedback loop where Cyclin B1 is ubiquitinated and tagged for destruction during the metaphase/anaphase transition (Pines and Hunter, 1991). The activation of the APC/C^CDC20^ and the subsequent decrease in Cyclin B1 protein levels results in a chain of events that resets the cell cycle in both daughter cells (Vigneron et al., 2018; Lara-Gonzalez et al., 2019) (see summary Figure 1B).

## Checkpoints Play a Critical Role in Homeostasis

Evolution has placed the cell cycle at the heart of monitoring the condition of the intrinsic and extrinsic environment of the cell. Select windows of time, or checkpoints, during cell cycle progression have been characterized to uniquely monitor for different forms of adverse events. There are five well characterized checkpoints: G1 phase of the cell cycle hosts the restriction checkpoint which determines whether a cell is suited to commit to DNA synthesis, S phase has a less well studied checkpoint critical for ensuring that re-replication cannot occur and to monitor for replication stress, at the boundary of G2 to M phase is the DNA damage checkpoint, mid-mitosis has the spindle assembly checkpoint to monitor the integrity of DNA prior to cell division and finally Aurora B controls cytokinesis through the abscission checkpoint.

**FIGURE 2:**
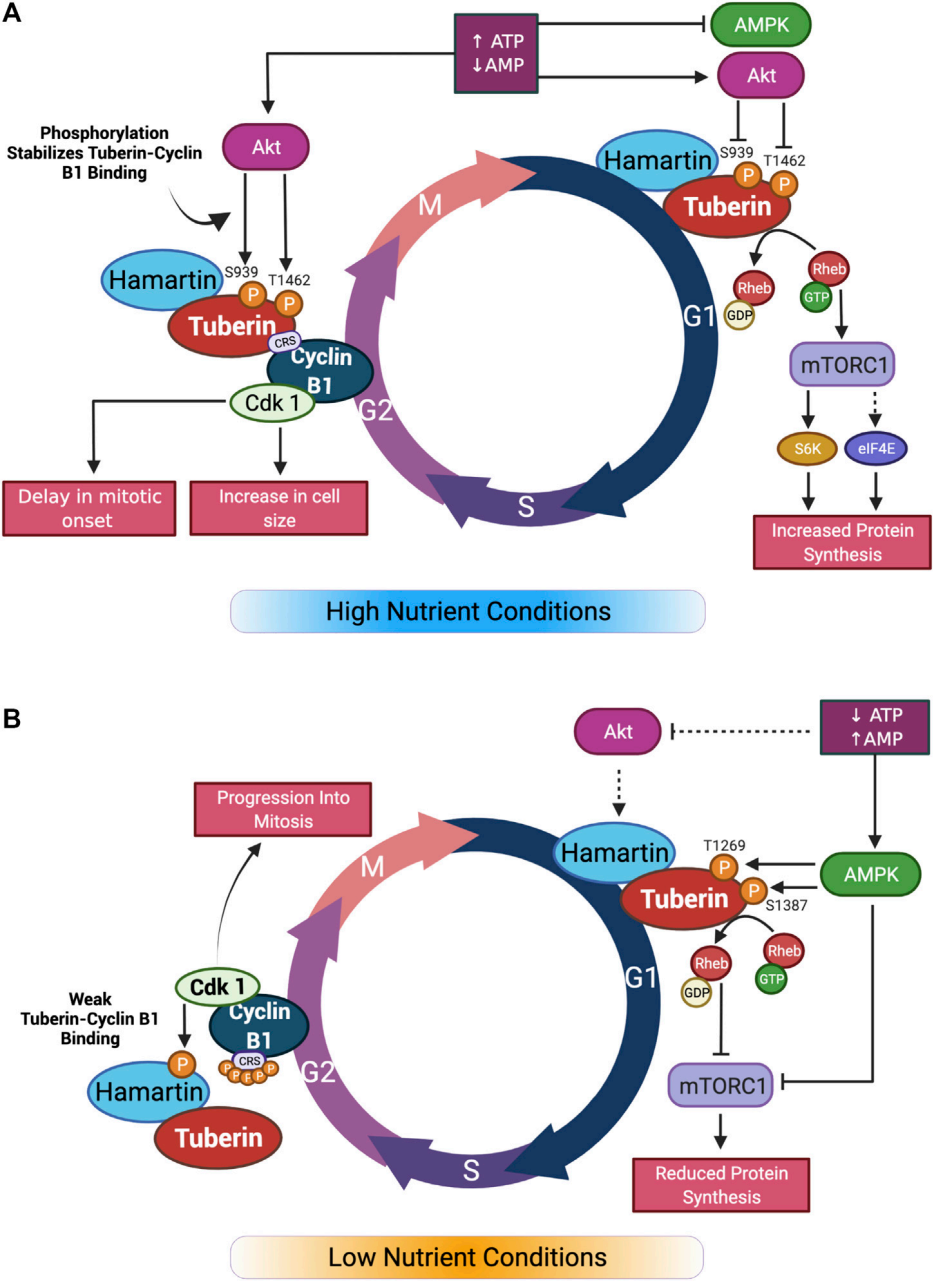
Graphical summary of nutrient effect on Tuberin-Cyclin B1 binding and cell cycle progression. **(A)** In high nutrient conditions, Akt inhibition of Tuberin through phosphorylation leads to activation of mTORC1 and increase in protein synthesis in G1. In G2 phase of the cell cycle, Tuberin-Cyclin B1 binding is increased by the same phosphorylation sites. This results in a delay in mitotic onset and an increase in cell size. **(B)** In low nutrient conditions, Akt does not phosphorylate Tuberin resulting in inhibition of mTORC1 and reduced protein synthesis in G1. In G2, the affinity of Tuberin binding to Cyclin B1 is reduced, resulting in a shortening of G2 and an increase in mitotic onset. Adapted from (Fidalgo da Silva et al., 2019). Created with BioRender.com.

The G1 restriction point is the most well studied and is a pivotal go: no-go point for the cell ensuring that adequate nutrients and growth factors are available to support the cell moving through a complete cycle of division (Bohnsack and Hirschi, 2004). As one example, under adequate nutrient conditions, Akt and Erk signaling are primary kinases connecting extracellular stimuli to the mammalian target of rapamycin 1 (mTORC1) to initiate protein synthesis, as well as activating or repressing the transcription of genes promoting progression through the cell cycle (Chambard et al., 2007; Xu et al., 2012) (Figure 2A).

Under adverse conditions the activation of tumor suppressors play an important role in halting cell cycle progression, primarily through the G1/S restriction point but also through the G2/M DNA damage checkpoint (Figure 1). p53 is the most widely studied transcription factor in this role, known to be mutated or deleted in over 50% of human cancers and implicated in a host of growth disorders (Rizzotto et al., 2021). One key downstream target of p53 is the CDK inhibitor p21^Cip1^, which functions to directly bind and inhibit the G1 CDK complexes, Cyclin E-CDK2 and Cyclin A-CDK2 (Xiong et al., 1993). p53 also plays a critical role in triggering the G2/M checkpoint during the DNA damage response (Taylor and Stark, 2001). Here, p53 transactivates key kinases, ATM and ATR, which dictate whether cells undergo cell cycle arrest, repair DNA or undergo apoptosis (Fridman and Lowe, 2003). p53 can also inhibit G2/M progression by transrepressing Cyclin B1 as well as repressing the phosphatase important for activating CDK1, Cdc25 (Innocente et al., 1999).

CDK inhibitors such as p21, p27, and p57 form one class of inhibitors capable of binding to Cyclins E, A, B, and CDKs 2 and 1 to inhibit cell proliferation. In mitogenic starved cells, quiescence can be achieved by elevating p21 and p27 and promoting their accumulation in the nucleus (Besson et al., 2008). The subsequent transactivation of the INK4a family of inhibitors can inhibit the early G1 CDKs, CDK4, and CDK6, leading to a state of permanent quiescence, referred to as senescence (Mirzayans et al., 2012; Liu et al., 2019).

Collectively, these checkpoints provide a rapid mechanism of putting the brakes on cell cycle progression in response to adverse environmental conditions or to respond to select developmental stimuli. There are a multitude of proteins described in the literature which provide a fine tuning of this control, this review will focus in on the TSC.

## TSC: A Regulator of Protein Synthesis, Cell Growth, Cell Adhesion, Transport, and Autophagy

The TSC is a large complex comprised of the proteins Tuberin (*TSC2*), Hamartin (*TSC1*) and TBC1 domain family member 7 (*TBC1D7*)*.* Research on the TSC began just under 200 years ago with the initial detection of benign skin lesions, that would later be determined to be caused by mutations in either *TSC1* or *TSC2* (Rayer and Bailliere 1835). Two centuries of research have revealed the important role of the TSC as a central regulator of tumor suppression via the ability to regulate many aspects of cell physiology. While each member of the complex plays a role in the stability and subcellular localization of the complex as a whole, the protein Tuberin contains functional domains responsible for the primary biological activity of the complex.

**FIGURE 3:**
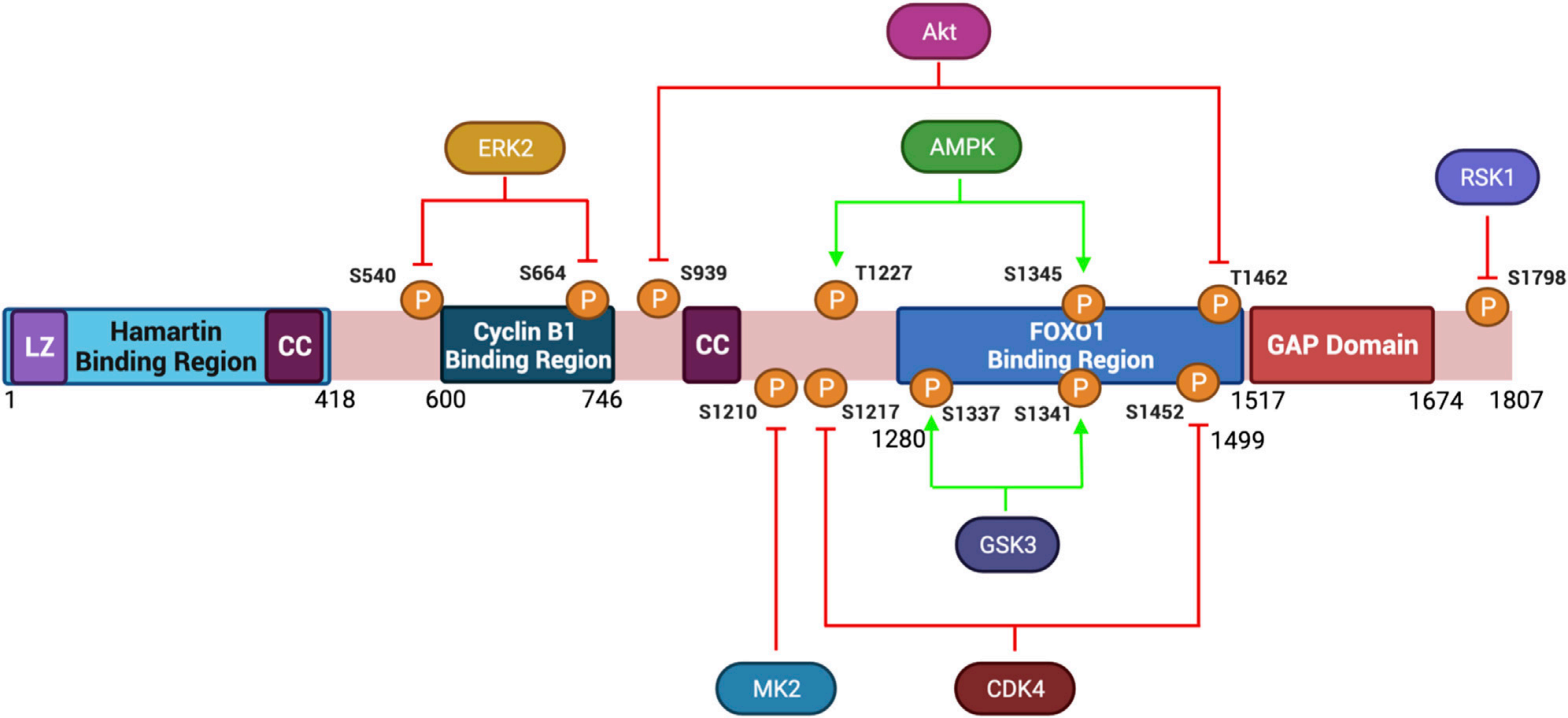
Protein Structure Diagram of Tuberin and key phosphorylation sites. Depicted is the primary structure of Tuberin, highlighting the binding regions to Hamartin (aa 1–418) and Cyclin B1 (aa 600–746), the GAP domain (aa 1,517–1,674), the leucine zipper motif (LZ), and the coiled-coil domain (CC). Upstream kinases of phosphorylation sites and modified residue numbers are shown with red arrows indicating inhibition and green arrows indicating activation of Tuberin activity. Created with BioRender.com.

Tuberin is found on chromosome 16 (16p13.3) and is a large 1807 amino acid protein with a molecular weight of 180 KDa (European Chromosome 16 Tuberous Sclerosis Consortium, 1993). The mRNA transcript harbors 41 exons and produces six isoforms (Sampson, 2003). Isoform 5 is the most frequently occurring and has a 1784 bp product resulting from the excision of exon 25 and 31 (Krymskaya, 2003; Ota et al., 2004). Tuberin contains several important structural and functional domains including two small coiled coil domains (aa 346–371; aa 1008–1021), a leucine zipper motive (aa 81–98), and a C-terminal GTPase-activating protein (GAP) domain (aa 1517–1674) (Rubinfeld et al., 1991, 1992; Hansmann et al., 2020) (Figure 3). As a part of the TSC, Tuberin accumulates in select subcellular locations including the nucleus, lysosome and mitochondria dependent on the cell cycle phase, nutrient availability and cellular stress (Clements et al., 2007; Demetriades et al., 2014) (Wienecke et al., 1996; Lou et al., 2001; Astrinidis and Henske, 2005).

The TSC integrates extracellular information regarding nutrient and growth factor levels and processes this to downstream signaling to ultimately regulate anabolic cellular processes, such as protein and lipid synthesis and ribosome biogenesis (Huang and Manning, 2008; Orlova and Crino, 2010). It does this primarily through the activity of the GAP domain in Tuberin, which stimulates the auto-hydrolysis of the Ras Homolog Enriched in Brain (Rheb)-GTP, converting it to the inactive Rheb-GDP (Inoki et al., 2002, 2003). When in an active form, Rheb-GTP releases an inhibitory protein, FK506-binding protein 38 (FKBP38), from the mTORC1 (Bai et al., 2007) (Rosner et al., 2003). mTORC1 is comprised of the proteins mTOR, Deptor (DEP-domain containing mTOR-interacting protein), Raptor (regulatory associated protein of mTOR), PRAS40 (proline-rich Akt substrate 40 kDa), and mLST8 (mammalian lethal with sec-13) (Yip et al., 2010; Catena and Fanciulli, 2017). mTORC1 has multiple roles in the cell, of which the most prominent is the initiation of protein synthesis. Activation of the mTORC1 complex leads to the phosphorylation of several downstream targets, the best characterized are S6 kinase 1 (p70 S6K1) and 4E binding protein 1 (4E-BP1) (Loewith et al., 2002). Phosphorylated p70 S6K1 serves as an activating kinase toward the S6 ribosomal protein to increase protein synthesis (Dufner and Thomas, 1999; Ruvinsky and Meyuhas, 2006). In the non-phosphorylated state, 4E-BP1 binds to and inhibits the eukaryotic initiation factor 4E (eIF4E) (Gingras et al., 1998, 2001). However, mTORC1-dependent phosphorylation inhibits 4E-BP1, resulting in the release of eIF4E from 4E-BP1. This then permits the initiation of cap-dependent translation, leading to an increase in protein translation, supporting cell growth. Hence, Tuberin indirectly inhibits protein synthesis and cell growth.

Tuberin depends heavily on post-translational modification directed by extracellular cues. Akt signaling for example leads to phosphorylation of Tuberin at S939 and T1462 disrupting the TSC complex formation thereby preventing Tuberin from hydrolyzing Rheb-GTP and supporting an active mTORC1 complex (Inoki et al., 2002). The subcellular localization of Tuberin may also be regulated by Akt-phosphorylation (Rosner et al., 2007b). In cycling cells, where Akt is active, Tuberin is predominantly in the cytoplasm, conversely under low energy/nutrient conditions Tuberin is predominantly nuclear. In addition to Akt, several other kinases phosphorylate Tuberin and inhibit TSC activity towards Rheb (Figure 3). Among these are Erk, which phosphorylates Tuberin at S540 and S664, and RSK1 which phosphorylates Tuberin at S1798 (Roux et al., 2004; Ma et al., 2005). It has also been observed that DAPK binds to Tuberin and promotes the dissociation of the TSC complex, details remain to be resolved for this specific kinase (Stevens et al., 2009). FOXO1 can also directly bind to Tuberin at residues 1280–1499 promoting the dissociation of TSC (Cao et al., 2006). Post-translational modifications of Tuberin can also support an active protein conformation, thereby inhibiting mTORC1. This can occur via the phosphorylation of Tuberin by the AMP-activated protein kinase (AMPK) on T1227 and S1345, as well as by the serine/threonine kinase GSK3β at S1337 and S1341, each of these modifications tend to activate Tuberin at times of low nutrient, growth factor and energy conditions (Inoki et al., 2003; Shaw et al., 2004; Huang and Manning, 2008; Orlova and Crino, 2010). During energy deprivation, AMPK phosphorylates Tuberin to inhibit translation, reduce cell size and protect the cells from apoptosis (Inoki et al., 2003). Collectively, Tuberin can be viewed as the integration point whereby extracellular signaling dictates outcomes in cell size.

In addition to protein synthesis and cell size, the TSC controls cell adhesion, cell spreading and cell migration. A loss of Tuberin protein results in increased cell migration, and this phenotype is dependent upon an upregulation of the α1β1-integrin receptor (Moir et al., 2012). Others have also found that loss of the TSC supports cell migration and have found that this is dependent on signaling through mTORC1 and mTORC2 (mammalian target of rapamycin 2) (Goncharova et al., 2004, 2014). The loss of Tuberin promotes stress fiber assembly via a mechanism involving the inhibition of Rac1 (Goncharova et al., 2004) and the activation of RhoA (Byrne et al., 2016). Other data shows that Tuberin has important functions at the cell membrane. Tuberin is required for the transport of Caveolin and the Vesicular Stomatitis Virus Glycoprotein (VSVG) to the membrane through the Golgi apparatus (Jones et al., 2004; Jiang and Yeung, 2006). In *TSC2* knock-out cells, these proteins fail to be transported and remain as punctate vesicles in the cytoplasm due to microtubule disorganization. Trafficking of glucose transporters and glucose uptake is also regulated by the Tuberin/mTORC1 pathway (Jiang et al., 2008).

A link between the TSC and apoptosis is also observed via the *TSC2/TSC1* knock-out cells. During glucose deprivation, these cells have elevated p53 translation due to the lack of mTORC1 inhibition and thereby rapidly undergo apoptosis (Choo et al., 2010). Tuberin has also been implicated in the regulation of DNA-damage induced autophagy (Liu et al., 2018). DNA damage triggers translocation of Tuberin to the lysosome thereby prompting the inactivation of Rheb and downstream inhibition of mTORC1. Similarly, autophagy induced due to low energy/nutrients levels can be initiated through AMPK phosphorylation of Tuberin and subsequent mTORC1 inhibition (Kim et al., 2011), but it can also be triggered independent of Tuberin via direct phosphorylation of Raptor by AMPK (Gwinn et al., 2008). The roles for Tuberin in autophagy appear to be important in normal development and to also be implicated in disease states such as TSC and cancer (Parkhitko et al., 2011; Reis et al., 2021).

## Tuberin and the Cell Cycle

While less well studied, Tuberin regulates the core cell cycle machinery in both mTORC1- dependent and independent manners. The interface with the cell cycle supports a mechanism by which the fitness of the cell can be integrated with decisions to grow and divide. In a seminal *Cell* publication Ito and Rubin used *Drosophila* cells harboring mutations in the gene homologous to *TSC2*, *gigas*, and found that the cells were enlarged and repeated S phase with a defect in the ability to enter into M phase (Ito and Rubin, 1999). These results were the first showing a TSC-dependent link between cell growth and cell cycle progression.

### Role of Tuberin in the G1/S Transition

The transcription factor c-Myc is a potent activator of cell cycle progression, upregulating G1 cyclin proteins and repressing the transcription of CDK inhibitors such as p27 (Hydbring et al., 2017). One mechanism by which Tuberin inhibits the cell cycle under low nutrient conditions, is via an indirect repression of c-Myc expression (Bretones et al., 2015). Tuberin inhibits mTORC1 to repress the translation of c-Myc (Schmidt et al., 2009; Pourdehnad et al., 2013; Csibi et al., 2014). Additionally, Tuberin has been shown to inhibit B-Raf/Erk signaling cascades in Quinol-thioether-transformed renal epithelial cells (Yoon et al., 2002, 2004), thereby indirectly inhibiting the transactivation of c-Myc due to the ability of Tuberin to convert Rap1-GTP into Rap1-GDP (Yoon et al., 2004; Chambard et al., 2007). It is notable that Rheb has been shown to inhibit Raf signaling (Im et al., 2002; Karbowniczek et al., 2006), hence the role of Tuberin on these signaling pathways may be context specific. Furthermore, Tuberin can bind to the β−catenin destruction complex and promote degradation of β−catenin, a protein that acts in a positive feedback loop to induce c-Myc expression (Mak et al., 2003). Interestingly, under high nutrient conditions, or with mitogenic stimulation, activated c-Myc directly represses Tuberin expression, providing a feedback loop that supports cell proliferation (Schmidt et al., 2009). Collectively these data support that Tuberin and c-Myc have opposing roles in the regulation of cell cycle progression through G1/S.

Tuberin can also be phosphorylated directly by the early G1 CDK, CDK4/6 on S1217, and S1452 (Romero-Pozuelo et al., 2020). This modification prevents the inactivation of Rheb, thereby blocking Tuberin-mediated inhibition of mTORC1 and increasing cell growth. Hence, in G1 phase of the cell cycle under favorable conditions Tuberin is actively inhibited to permit cell growth.

Through several different bodies of work Rosner et al. have shown that Tuberin can potentiate the activity of the CDK inhibitor p27 (Rosner et al., 2006; 2007a; 2007b). p27 can be bound by 14-3-3 and localized to the cytoplasm where it can be subject to Skp2-mediated degradation, thereby preventing the inhibition of CDKs (Rosner and Hengstschläger, 2004). Under serum starvation conditions Akt does not phosphorylate Tuberin, a post-translational status which allows Tuberin to interact with p27 and interfere with 14-3-3 interaction (Rosner et al., 2007a). The details of the interaction between Tuberin and p27 remain to be fully resolved, however it has been shown that Tuberin facilitates the nuclear localization of p27 and prevents Skp2-mediated degradation, collectively permitting the inhibition of cyclin-CDK complexes and enforcing the G1/S checkpoint. Upon stimulation with mitogens, Akt signaling is activated which phosphorylates p27 on T157 in the NLS (Shin et al., 2002; Rosner et al., 2007a). This modification dissociates the Tuberin-p27 complex, p27 is again bound by 14-3-3 and localized to the cytoplasm to be targeted for degradation. This allows cyclin-CDK activation and cell cycle progression through G1.

### Role of Tuberin in the G2/M Transition

Cells need to grow to two times their size prior to division to retain homeostatic balance over time, the details of how this is achieved remains unresolved. Petersen and Nurse previously described a link between the mTORC1 pathway and mitotic regulation in *S. Pombe* in response to nutrient availability (Petersen and Nurse, 2007). In fission yeast they demonstrated that low nitrogen conditions, or Rapamycin treatment, resulted in a faster movement into mitosis. They concluded that this was dependent upon the activity of Plk1. The work of others has demonstrated that Plks and CDK1 phosphorylate Hamartin during G2/M and mitosis connecting cell division and protein synthesis (Astrinidis et al., 2003; Li et al., 2018). Hamartin is phosphorylated by CDK1 at T417, S584, and T1047 during the G2/M phase of the cell cycle, and although Hamartin is still able to form a complex with Tuberin, Hamartin regulates the p70 S6K activity allowing increased protein synthesis and cellular growth during late mitosis/early G1 phase. While the exact mechanism is not clear, CDK1 is known to phosphorylate S6K on S70 (Shah et al., 2003), it is possible Hamartin releases the inhibitory CDK1 phosphorylation of p70 S6K. During mitosis, Hamartin is phosphorylated by Plk1 at S467 and S578, the phosphorylation of these residues destabilizes Hamartin and promotes the dissociation of the Tuberin-Hamartin complex. (Li et al., 2018). It is also known that CDK1 directly phosphorylates Raptor during mitosis promoting dissociation from the lysosome and subsequent inhibition of mTORC1 (Odle et al., 2021). TACC3, a kinase responsible for centrosome activity and microtubule assembly during cell division (Gergely et al., 2000; Peset and Vernos, 2008), phosphorylates Tuberin on S939 (Gómez-Baldó et al., 2010). This alteration localizes Tuberin to the mitotic apparatus and cytokinetic structures during mitosis promoting proper cytokinetic abscission.

A direct role for Tuberin in the regulation of the G2/M transition has also been described. Our lab and others have characterized a direct interaction between Tuberin and Cyclin B1 in mammalian cells (Catania et al., 2001; Fidalgo da Silva et al., 2011, 2016, 2019). We have resolved that Tuberin contains a Cyclin B1 binding domain between exon 16 and 17 (aa 600–746) and have described that this functions to retain Cyclin B1 in the cytoplasm during the G2/M transition, delaying the mitotic onset. Additionally, the Tuberin-Cyclin B1 complex is formed in the absence of the Tuberin GAP domain, demonstrating that the observed effects are independent of mTORC1 (Jones et al., 1999; Cheadle et al., 2000; Fidalgo da Silva et al., 2011).

The binding of Tuberin to Cyclin B1 is also sensitive to the differential phosphorylation states in both Tuberin and Cyclin B1. Akt phosphorylation of Tuberin at S939 and T1462 for example, stabilizes the Tuberin-Cyclin B1 interaction, thereby retaining Cyclin B1 in the cytoplasm, delaying mitotic onset, and permitting cell growth (Fidalgo da Silva et al., 2019) (Figure 2A). In this work, flow cytometry analysis of cells synchronized at G1/S phase transition via double thymidine block and released through the cell cycle demonstrate that preventing Akt phosphorylation of Tuberin causes rapid progression of cells through G2 phase of the cell cycle and into mitosis, this same effect was observed when the cells were cultured in low serum conditions (Figure 2B). An ECFP-G2 reporter was created to study the timing of G2 more specifically and this system further confirmed that low serum conditions support a shortened G2 phase and more rapid entry into mitosis (Fidalgo da Silva et al., 2016). It has also been shown that the phosphorylation status of Cyclin B1 within the CRS region plays a role in the binding to Tuberin (Fidalgo da Silva et al., 2011). Here, a stronger complex is formed when the Cyclin B1-CRS region is unphosphorylated, supporting retention of Cyclin B1 in the cytoplasm. A current preprint supports that early phosphorylation of the CRS region on S126 by CDK1 reduces the affinity of binding between Tuberin and Cyclin B1 (Dare-Shih et al., 2021).

**FIGURE 4:**
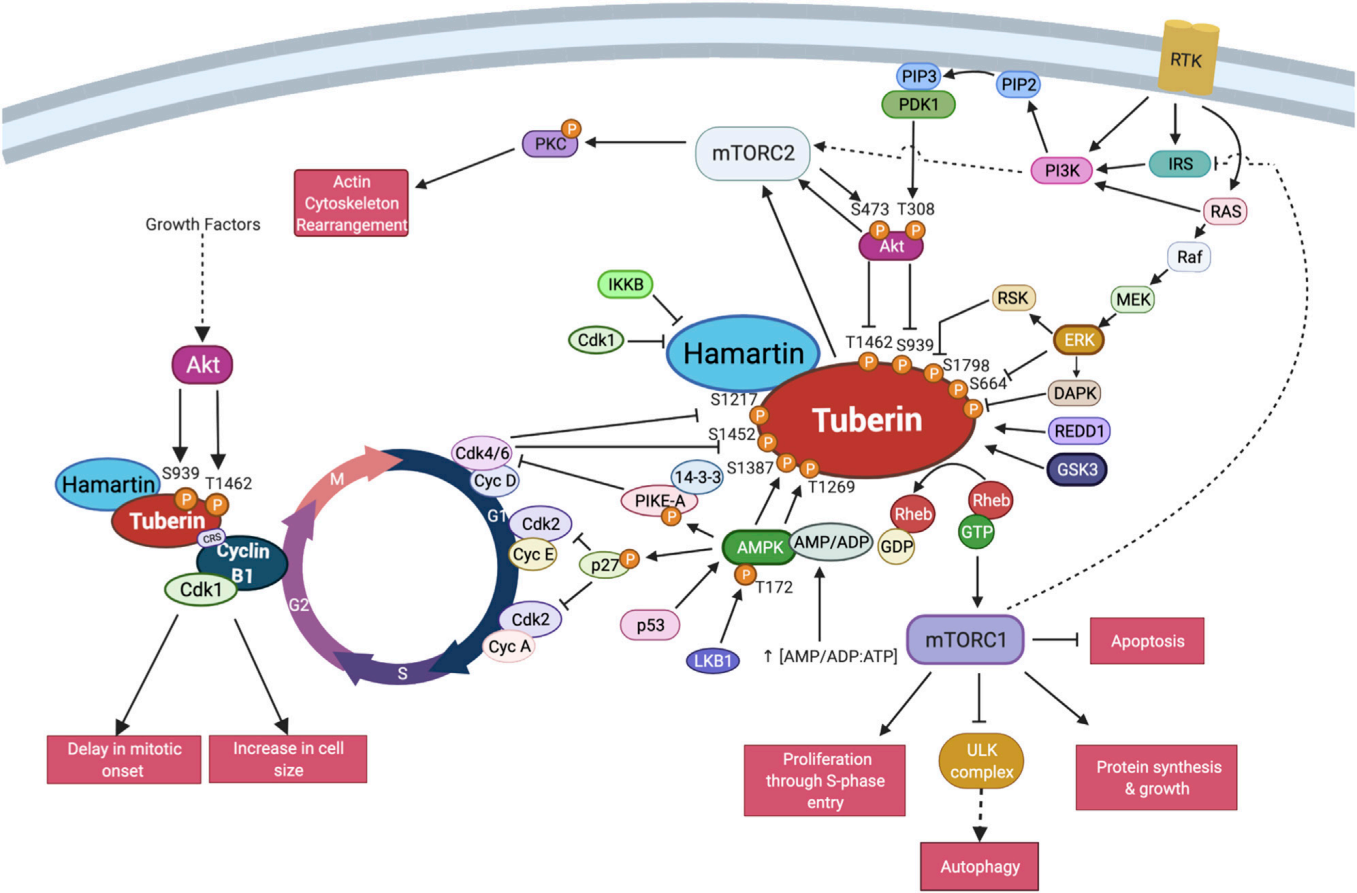
TSC interactions with the core cell cycle machinery. Schema of the described interactions between Tuberin-Hamartin and the cell cycle proteins in select regions of the cell division cycle. This includes post-translational modifications on Tuberin known to impact cell cycle interactions. Created with BioRender.com.

Collectively several bodies of data support the presence of a novel serum sensitive early G2 checkpoint dependent on interactions between Tuberin and Cyclin B1 (see summary Figure 4). Further resolving the details of this interaction will reveal mechanisms by which a cell controls cell division and cell growth contingent on nutrient availability.

## Functional Consequences of Tuberin Mutations in the Cell Cycle

Mutations in either *TSC1* or *TSC2* disrupt the function of the TSC causing cell growth and cell division defects, resulting in pathogenesis associated with the TSC. TSC disorder affects 1 in 6,000 live births annually, with an estimated 1.5 million individuals living with TSC worldwide (European Chromosome 16 Tuberous Sclerosis Consortium, 1993; Maheshwar et al., 1997; Jones et al., 1999; Curatolo et al., 2008; Grajkowska et al., 2010; Wataya-Kaneda et al., 2013). The phenotypes of patients with TSC range widely from dermalogical manifestations, such as facial angiofibroma to manifestations affecting the brain, such as cortical tubers, subependymal nodules (SENs), or subependymal giant cell astrocytomas (SEGA) can cause blood vessel obstruction and cerebrospinal fluid accumulation, which can be lethal (Henske et al., 1997; Orlova and Crino, 2010; Han and Sahin, 2011; Kohrman, 2012). The central nervous system is affected in nearly 50% of TSC patients and can result in forms of autism, epilepsy (in 60–90% of cases), mild to severe learning difficulties, and behavioural disorders such as Attention Deficit Hyperactive Disorder (de Vries and Howe, 2007; Ehninger et al., 2009; Numis et al., 2011). Outside of the central nervous system angiomyolipomas developing within the kidney of TSC patients may lead to kidney failure and impaired function, haemorrhaging and possible development of carcinomas (Rosner et al., 2008; Orlova and Crino, 2010). Cardiac hypertrophy and type 2 diabetes have also been linked to TSC via AMPK and p70S6K hyper-activation as a result of Tuberin malfunction (Orlova and Crino, 2010). Primary lymphedema leading to swelling of the extremities may be an early diagnostic in the detection of TSC for a small percentage (4%) of affected patients (Geffrey et al., 2014; Klinner et al., 2020). In addition to the role of Tuberin in the benign tumours of TSC patients, Tuberin mutations have also been found to cooperate with oncogenic mutations aiding in the initiation and progression of a number of malignant cancers affecting the brain (medulloblastoma), lung, kidney (renal cell carcinomas), and breast (Dabora et al., 2001; O’Callaghan et al., 2004; Bhatia et al., 2009; Franz et al., 2010; Orlova and Crino, 2010). The range of diseases and phenotypes associated with mutations in *TSC1*/*TSC2* can be attributed to the fact that mutations tend to span the entire length of these large genes and this speaks to the complexity of their protein products. Understanding why different mutations in the TSC cause different phenotypes will reveal important biology, including how this complex communicates with cell growth and division.

TSC gene mutations arise by two routes: genetic inheritance, with *TSC1* or *TSC2* mutations being passed to patients genetically through existing familial mutations or through sporadic, *de novo* mutations, which occur during embryonic development. *De novo* mutations have been observed to underlie the greater majority of TSC cases, with approximately 60–70% of TSC cases showing no genetic inheritance (Jones et al., 1997, 1999; Uysal and Sahin, 2020). *TSC2* mutations are nearly five times more common than mutations in *TSC1*, and patients present TSC2 mutations are nearly five times more common than mutations in TSC1, and patients present with more severe phenotypes (Jones et al., 1999; Astrinidis and Henske, 2005; Sancak et al., 2005). Loss of heterozygosity occurs within the somatic cells of TSC patients and is often cited as a likely cause of tumor development associated with TSC, as inactivation of both alleles appears to be required for lesion formation (Crino et al., 2006). In agreement with Knudson’s tumor suppressor model, this loss of heterozygosity occurs due to second-hit mutation events, which commonly take the form of large deletions involving the loss of surrounding loci (Knudson, 1971). These data collectively support the role of the Tuberin protein as a classic tumour suppressor within cell cycle regulation.

For many years, mutational studies have been conducted to investigate the spectrum of mutations observed in TSC patients. *TSC1* mutations predominantly occur by nonsense or frame shift mutations, leading to premature protein truncation upon translation. *TSC2* mutations on the other hand, present a broader spectrum including frameshift, missense, nonsense, in-frame deletions and splice mutations (Jones et al., 1999). More than 200 *TSC1* and 700 *TSC2* allelic variants have been identified to date (Kwiatkowska et al., 1998; Jones et al., 1999; Niida et al., 1999; van Slegtenhorst et al., 1999). Many TSC patients have presented an elevated frequency of missense mutations in the *TSC2* gene, occurring at R611Q/W (exon 16), P675L (exon 38) and an 18-bp in frame deletion in exon 40 (Sancak et al., 2005). Missense mutations in *TSC2* also have been identificated in the GAP domain, between exons 35 and 39. Large genomic deletions and rearrangements in *TSC2* can affect the adjacent *PKD2* gene, causing early-onset polycystic kidney disease (Crino et al., 2006). Dissecting the consequence of specific alterations to *TSC2* may shine light on many of the cell biology functions of the Tuberin protein, including how it integrates between cell growth and cell division.

**FIGURE 5:**
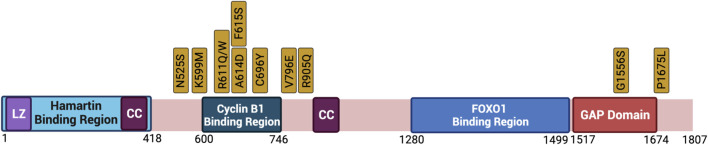
Clinically relevant missense mutations within the *TSC2* gene that abrogate the function of the protein. Depicted is the primary structure of Tuberin, highlighting the binding regions to Hamartin (aa 1–418) and Cyclin B1 (aa 600–746), the GAP domain (aa 1517–1674), the leucine zipper motif (LZ), and the coiled-coil domain (CC). Golden boxes show clinical mutations. Created with BioRender.com.

Data has shown that clinically relevant missense mutations within the *TSC2* gene abrogate the function of the protein. Tuberin mutations such as R611Q, R611W, A614D, C696Y, F615S, and V796E disrupt the chaperone function of Tuberin with respect to Hamartin and interfere with Akt phosphorylation of Tuberin (Aicher et al., 2001; Nellist et al., 2001, 2005; Astrinidis and Henske, 2005). Importantly, these residues are located in a mutational hot-spot in the Cyclin B1 binding domain (Figure 5). The consequence of decreased TSC complex formation is the increase Rheb-GTPase activity and S6K phosphorylation when these Tuberin variants are compared to Tuberin wild type (Nellist et al., 2001). It has been demonstrated that C696Y mutations disrupt the binding of Tuberin to Cyclin B1 as well, and abolish the G2/M transition and cell size regulation by Tuberin (Fidalgo da Silva et al., 2011, 2019). Mutations residing outside of the Cyclin B1 binding domain, such as N525S, K599M, R905Q, and G1556S along with others, do not elicit the same effects and disease severity (Nellist et al., 2005).

## Summation

The protein Tuberin integrates environmental stimuli to regulate a host of physiological responses of a cell, including cell growth and division. While a great deal of work has occurred to study how these pathways regulate protein synthesis, data supports that this also provides a key link to the cell division cycle and overall cell homeostasis. Details regarding the physical interaction between Tuberin and p27 remain to be resolved. How Tuberin facilitates the nuclear movement of Cyclin B1 and the onset of mitosis also requires more research. Whether either of these processes require the other proteins within the TSC and whether mTORC1/2 and the protein translational machinery can regulate these interactions to provide cross talk is an intriguing idea that would reinforce the connection between cell growth and cell division.

Much of the current study of TSC involves only full knock-outs of *TSC1* or *TSC2,* an approach which fails to shine light on the nuances of the normal biology that hinges on these large important proteins. Naturally occurring disease models hold many answers that remain to be fully explored. The advent of Crispr-Cas technology has changed the pace at which the field can dissect select *TSC1/TSC2* mutations. A great deal more work is required to reveal the cell biology behind each of these mutations. History in this field has shown that this will require separate focus in different cell types under different intrinsic and extrinsic conditions. Hence, while the amount of work needed is still great, the path forward is becoming more clear and stands to provide fundamental knowledge which underlies important aspects of the growth, development, and homeostasis of all organisms.
